# Inhibitory activity of monoacylglycerols on biofilm formation in *Aeromonas hydrophila*, *Streptococcus mutans*, *Xanthomonas oryzae*, and *Yersinia enterocolitica*

**DOI:** 10.1186/s40064-016-3182-5

**Published:** 2016-09-09

**Authors:** Youngseok Ham, Tae-Jong Kim

**Affiliations:** Department of Forest Products and Biotechnology, College of Forest Science, Kookmin University, 77 Jeongneung-ro, Seongbuk-gu, Seoul, 02707 Korea

**Keywords:** *Aeromonas hydrophila*, *Streptococcus mutans*, *Xanthomonas oryzae*, *Yersinia enterocolitica*, Biofilm formation, Monoacylglycerol

## Abstract

Biofilm provides a bacterial hiding place by forming a physical barrier and causing physiological changes in cells. The elimination of biofilm is the main goal of hygiene. Chemicals that are inhibitory to biofilm formation have been developed for use in food, personal hygiene products, and medical instruments. Monoacylglycerols are recognized as safe and are used in food as emulsifiers. In this study, the inhibitory activity of monoacylglycerols on bacterial biofilm formation was evaluated systematically with four bacterial strains, *Aeromonas hydrophila*, *Streptococcus mutans*, *Xanthomonas oryzae*, and *Yersinia enterocolitica*. Monoacylglycerols with two specific lengths of fatty acid moiety, monolaurin and monobehenin, were found to have strong inhibitory activity toward bacterial biofilm formation of *S*. *mutans*, *X*. *oryzae*, and *Y*. *enterocolitica* in a strain specific manner. First, this result suggested that biofilm formation was not inhibited by the detergent characteristics of monoacylglycerols. This suggestion was supported by the inhibitory action of monolaurin on biofilm development but not on the initial cell attachment of *Y*. *enterocolitica* in flow cytometric observation. Second, it was also suggested that two distinct response mechanisms to monoacylglycerols existed in bacteria. The existence of these two inhibitory response mechanisms was bacterial strain specific.

**Electronic supplementary material:**

The online version of this article (doi:10.1186/s40064-016-3182-5) contains supplementary material, which is available to authorized users.

## Background

Bacterial biofilm is the primary source of bacterial persisting contamination. Without biofilm formation, bacteria can be easily washed away. Even before producing a mature biofilm, the condition for bacterial biofilm formation induces bacterial cells to stick together on a hard surface which is the initiation of biofilm formation and makes it difficult to remove bacteria during the process of biofilm formation. However, the function of biofilm as a contamination source is not only due to the difficulty of removing bacteria physically but also due to the physiological changes in cells that increase their resistance to physical, chemical, and biological stresses (Park et al. 2015; Parsek and Singh 2003).

Monoacylglycerols are organic compounds which are consisted with glycerol linked to one fatty acid via an ester bond. They exist in some seeds naturally and can be synthesized in cells as intermediate compounds in the process of synthesis and hydrolysis of triacylglycerols. Monoacylglycerols are generally recognized as safe by the US Food and Drug Administration, and are used as emulsifiers in commercial food products. Some monoacylglycerols inhibit bacterial growth and also change bacterial physiology as follows. Monocaprylin is toxic to *Cronobacter sakazakii* and *Cronobacter malonaticus* (Marounek et al. 2012). Monocaprin has shown antimicrobial activities against *Streptococcus mutans* (Ósk Thorgeirsdóttir et al. 2006). Monolaurin inhibited growth and reduced exotoxin production in *Staphylococcus aureus* (Schlievert et al. 1992) and also inhibited the expression of virulence factors in *S. aureus* (Ruzin and Novick 2000). In addition, it inhibited the production of virulence factors in *Bacillus anthracis* (Vetter and Schlievert 2005), and repressed the induction of vancomycin resistance in *Enterococcus faecalis* (Ruzin and Novick 1998). Monoacylglycerols inhibited the growth of food-borne pathogens or spoilage bacteria strains, and monolaurin especially showed strong inhibition of the cell growth of five strains: *Bacillus cereus*, *B. subtilis*, *E. faecalis*, *Micrococcus luteus*, and *S. aureus* (Buňková et al. 2011). Monocaprin, monolaurin, and monomyristin inhibited the growth of several Gram-positive strains and monolaurin had the most effective inhibitory activity (Batovska et al. 2009). In addition, monolaurin (Chavant et al. 2004; Oh and Marshall 1996) and its formulations (Hess et al. 2014; Lester and Simmonds 2012; Rouse et al. 2005) showed a bactericidal activity on cells in biofilm.

In addition to their bactericidal activity, the inhibition of biofilm formation by monoacylglycerols can be expected because of their surfactant properties (Thanomsub et al. 2004). However, the inhibitory efficacy of monoacylglycerols on biofilm formation has not been well evaluated. Monolaurin inhibited biofilm formation in *S*. *aureus* and *Haemophilus influenzae* in addition to their bactericidal activity (Schlievert and Peterson 2012). In this study, the inhibitory activity of monoacylglycerols against biofilm formation in four bacterial strains, *Aeromonas hydrophila*, *S. mutans*, *Yersinia enterocolitica*, and *Xanthomonas oryzae*, was evaluated. *A*. *hydrophila* is a major pathogen of fish and amphibians (Khalil et al. 2013; McGarey et al. 1991). *S*. *mutans* exists in the human oral cavity (Struzycka 2014) and is a major contributor to the development of dental caries (Forssten et al. 2010). *Y*. *enterocolitica* is an opportunistic pathogen that causes yersiniosis (Koornhof et al. 1999; Smego et al. 1999), which is a serious problem in infected children under the age of one (Abdel-Haq et al. 2000). *X*. *oryzae* is a plant pathogen that causes bacterial blight in rice (Shen and Ronald 2002). Among these four bacterial strains, the inhibitory effect of monolaurin on biofilm formation in *S*. *mutans* was investigated, particularly as an oral hygiene compound.

## Methods

### Bacterial strains and culture media

*Aeromonas hydrophila* and *S. mutans* were obtained from the Research Institute of LG Household a Health Care Co., Ltd. (Daejeon, Korea). *X. oryzae* (KACC 10331) and *Y. enterocolitica* (KCCM 41657) were obtained from the Genebank Information Center in the Rural Development Administration (Jeonju, Korea) and Korean Culture Center of Microorganisms (Seoul, Korea), respectively.

YGC agar plates were made with 50 g/l of glucose, 5 g/l of yeast extract, 12.5 g/l of CaCO_3_, and 15 g/l of agar. M210 medium and XOM2 medium were prepared as previously described by Zhang et al. (2013) and Tsuge et al. (2002), respectively. LB agar plates were made with 25 g/l of LB broth (Becton, Dickinson, and Company, Franklin Lakes, NJ, USA) and 15 g/l of agar. TYE medium was made with 10 g/l of tryptone and 5 g/l of yeast extract. M63G medium (100 ml) was made with 20 ml of 5× minimal M63 medium (15 g/l of KH_2_PO_4_, 35 g/l of K_2_HPO_4_, 10 g/l of (NH_4_)_2_SO_4_, and 2.5 mg/l of FeSO_4_), 1 ml of 20 % glucose, 0.1 ml of 20 % MgSO_4_, and filled with water. BHI medium was made with 37 g/l of brain heart infusion broth (Becton, Dickinson, and Company). To make BHI agar plates, 15 g/l of agar was additionally added to BHI medium. To make BHI-S medium, 10 g/l of sucrose was additionally added to BHI medium (Beckloff et al. 2007; Coenye et al. 2007). Tryptone soy broth with yeast extract (TSBY) was prepared as previously described (Kirov et al. 2004).

### Monoacylglycerols

Monocaprylin (1-octanoyl-*rac*-glycerol, catalog number: M2265) and monocaprin (1-decanoyl-*rac*-glycerol, catalog number: M2140) were purchased from Sigma-Aldrich Korea Co. (Seoul, Korea). Monolaurin (1-lauroyl-*rac*-glycerol, catalog number: G0081) was purchased from Tokyo Chemical Industry Co., LTD (Tokyo, Japan). Monomyristin (1-myristoyl-*rac*-glycerol, catalog number: O-1335) and monopalmitin (1-palmitoyl-*rac*-glycerol, catalog number: O-1420) were purchased from Bachem AG (Bubendorf, Switzerland). Monostearin (1-stearoyl-*rac*-glycerol, catalog number: 43883) was purchased from Alfa Aesar (Seoul, Korea). Monoarachidin (1-arachidoyl-*rac*-glycerol, catalog number: 31-2000) and monobehenin (1-behenoyl-*rac*-glycerol, catalog number: 31-2200) were purchased from Indofine Chemical Company, Inc. (Hillsborough, NJ, USA).

### Biofilm formation in 96-well microplates

*Xanthomonas oryzae* stored at −80 °C was streaked on YGC agar plates and incubated at 28 °C for 2 days. A single colony was inoculated in 5 ml of M210 and incubated at 28 °C and 250 rpm for 2 days. The cell density of the culture was measured at A_600_. The cultured cells were inoculated in 100 μl of XOM2 media in 96-well polyvinyl chloride (PVC) microplates with 0.05 of A_600_ as the final cell concentration. The cells in the microplates were incubated at 28 °C for 24 h and the amount of biofilm was quantified using crystal violet.

*Yersinia enterocolitica* biofilm was produced according to a previously described method (Kim et al. 2008).

*Streptococcus mutans* biofilm was produced according to a method previously described for *X*. *oryzae* with some modifications for *S*. *mutans*. All cultures were cultivated at 37 °C. BHI agar plates were used to obtain a single colony from the stored strain and BHI medium was used to prepare a pre-culture for 1 day. BHI-S medium was used for biofilm formation in 96-well PVC microplates.

*Aeromonas hydrophila* biofilm was also produced according to a method previously described for *X*. *oryzae* with some modifications for *A*. *hydrophila*. All cultures were cultivated at 30 °C. LB agar plates were used to obtain a single colony from the stored strain for 1 day and LB medium was used to prepare a pre-culture for 1 day. TSBY medium was used for biofilm formation in 96-well PVC microplates.

### Cell density and bacterial biofilm quantification using crystal violet

After 24 h of biofilm formation in 96-well PVC microplates, optical density of each well at 595 nm was measured before quantitative analysis of biofilm using an Opsys MR microplate reader (Dynex Technologies, Chantilly, VA, USA). The cell density was correlated with the optical density at 595 nm. Quantitative analyses of biofilms were performed using the crystal violet assay as described in a previous study (Kim et al. 2008) with a change of all incubation times from 20 to 15 min. The optical density at 595 nm of each well was also measured using an Opsys MR microplate reader.

### Biofilm formation in continuous-culture flow cells

Biofilm formation of *S*. *mutans* in a flow cell system was observed as reported in a previous experiment with some modifications (Kim et al. 2008). The flow cells and the bubble traps were purchased from the Center for Biomedical Microbiology (Technical University of Denmark, Lyngby, Denmark). The flow cells were covered with microscope coverslips made from polyvinyl chloride. Media were fed using a peristaltic pump (Masterflex L/S Digital Drive EW-07523-90, Masterflex L/S 12-channel, 8-roller cartridge pump head EW-07519-25, Masterflex L/S small cartridges EW-07519-85, Cole-Parmer Instrument Company, LLC., Vernon Hills, IL USA).

First, monolaurin was dissolved in methanol and mixed with media in order to facilitate dissolution. The final concentration of monolaurin in BHI-S medium was 95 or 190 mg/l with 0.48 % methanol. *S*. *mutans* was incubated in 5 ml of BHI medium for 24 h at 37 °C without shaking. The cells were diluted to an OD_600_ of 0.15 with BHI-S media and inoculated in a 350 μl volume. The flow cells were incubated for 1 h with the coverslip side down. The flow cells were turned over and fed with media for 24 h at a speed of 13 ml/h according to a previous study (Wen et al. 2006). Biofilm formed on the coverslips was observed after 1 and 24 h of incubation using an Axio Scope.A1 microscope (Carl Zeiss Co., LTD., Seoul, Korea) with ZEN microscope software (Carl Zeiss Co., LTD.).

### Measuring the sterilization effect of monolaurin on *S*. *mutans* in a planktonic growth

The inhibitory activity of monolaurin on the growth of *S*. *mutans* was measured by the colony forming unit (CFU) method. The monolaurin was dissolved in 10 % methanol immediately before treatment. Monolaurin was tested with various concentrations ranging up to 190 mg/l. The cells were incubated in BHI broth at 37 °C for 24 h as a pre-culture. The cultured cells with 0.05 of A_600_ as a final cell concentration were inoculated into 2 ml BHI media with 1 % sucrose and the test concentration of monolaurin. Cells were additionally incubated at 37 °C for 24 h. The homogeneous culture solution (100 µl) was spread onto BHI agar plates after serial dilution. The spread plates were incubated at 37 °C for 48 h and the number of colonies was counted. All experiments were repeated three times at each concentration.

## Results and discussion

**Fig. 1:**
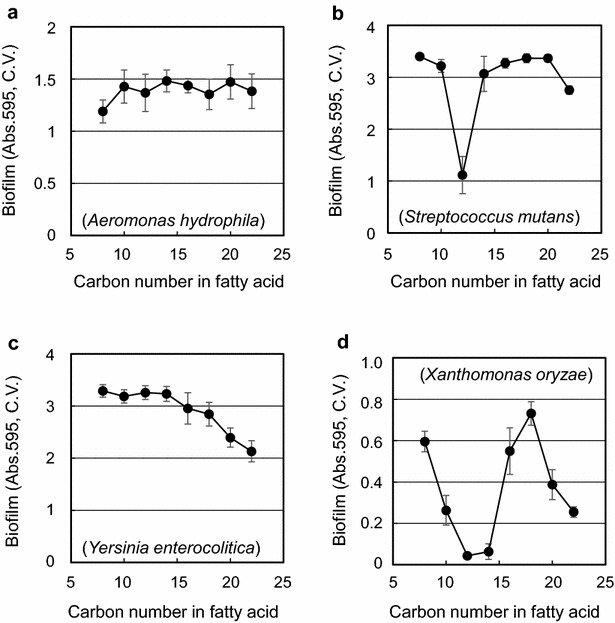
Effect of the fatty acid length of monoacylglycerols on biofilm formation by four bacterial strains, **a** *A*. *hydrophila*, **b** *S*. *mutans*, **c** *Y*. *enterocolitica*, and **d** *X*. *oryzae*. The concentration effect of each monoacylglycerol was first observed in Additional file 1: Fig. 1. Because the results obtained with 48 mg/l clearly demonstrated the effectiveness of monoacylglycerols on biofilm formation, we selected 48 mg/l treatment data and replotted only selected data here. The biofilm amount was evaluated with 1 % crystal violet. Data points are the average of eight experiments and are presented as mean ± SD

The effect of monoacylglycerols on biofilm formation was evaluated according to the carbon skeleton length of the fatty acid moiety using four bacterial strains, *A. hydrophila*, *S. mutans*, *X. oryzae*, and *Y. enterocolitica*. In the case of *A*. *hydrophila*, all tested monoacylglycerols did not change biofilm formation in the tested concentration range (Fig. 1a; Additional file 1: Fig. 1A). There was no effect of the tested monoacylglycerols on biofilm formation of *A*. *hydrophila*.

Monoacylglycerols clearly altered biofilm formation in *S*. *mutans* in a carbon skeleton length-specific manner (Fig. 1b; Additional file 1: Fig. 1B). Monocaprylin did not have any distinct inhibitory activity on biofilm formation. Monocaprin began to have inhibitory activity by 7 % with 48 mg/l (Additional file 1: Fig. 1B). Monolaurin had the strongest inhibitory activity, reducing biofilm formation by 66 % with 48 mg/l of monolaurin having a similar inhibitory effect on biofilm formation in *S*. *aureus* (Schlievert and Peterson 2012). Monomyristin began to relieve the inhibitory activity on biofilm formation and had similar inhibitory activity to that of monocaprin, 10 % inhibition with 48 mg/l. Monopalmitin, monostearin, and monoarachidin did not have any inhibitory activity, while the inhibitory activity of monobehenin was minor. To summarize the results, monolaurin had the strongest inhibitory activity on biofilm formation in *S*. *mutans*.

Biofilm formation of *Y*. *enterocolitica* was not inhibited by monocaprylin, monocaprin, monolaurin, or monomyristin but started to be inhibited by monopalmitin and longer monoacylglycerols (Fig. 1c; Additional file 1: Fig. 1C). The longer the carbon skeleton monoacylglycerols had, the stronger the inhibitory activity they had among monopalmitin and longer monoacylglycerols.

Monoacylglycerols inhibited the biofilm formation of *X*. *oryzae*, much like *S*. *mutans* but with stronger effects (Fig. 1d; Additional file 1: Fig. 1D). The strong inhibitory activity on biofilm formation was observed in two different ranges of carbon skeleton length. One range was with monolaurin and monomyristin and the other was with monobehenin.

**Fig. 2:**
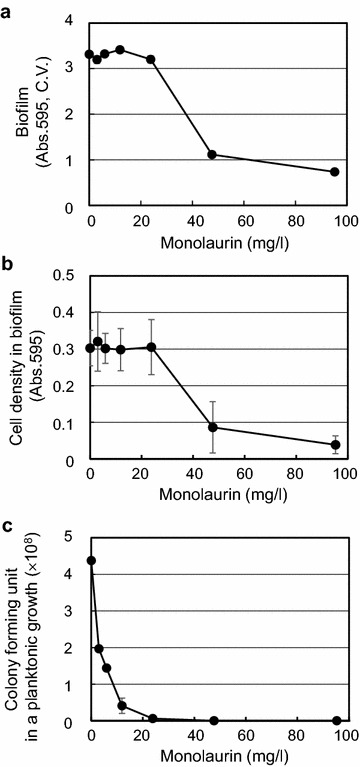
The inhibitory effects of monolaurin on *S*. *mutans*. The effects of monolaurin on **a** biofilm formation, **b** cell growth in biofilm, and **c** cell growth in a planktonic growth of *S*. *mutans* were evaluated

Because *S*. *mutans* is a major bacteria in dental plaque and its acid production causes tooth decay, the inhibitory activity of monoacylglycerols on biofilm formation in *S*. *mutans* was investigated more thoroughly than that of other strains in this study. The inhibitory activity of monolaurin on biofilm formation of *S*. *mutans* showed that biofilm formation was inhibited and the inhibitory effect was correlated with the monolaurin concentration above 24 mg/l (Fig. 2a). The cell growth during biofilm formation was inhibited in the same way (Fig. 2b). However, the inhibitory activity of monolaurin was different between cells in planktonic growth and cells in biofilm (Fig. 2b, c). Monolaurin at 3 mg/l strongly inhibited planktonic cell growth but the cell growth in biofilm was not inhibited up to 24 mg/l monolaurin. This result clearly demonstrated the increased resistance of *S*. *mutans* in biofilm to the monolaurin treatment. In the case of *Y*. *enterocolitica* and *X*. *oryzae*, there was no strong correlation between biofilm formation and cell growth when cells were treated with monoacylglycerols (Additional file 1: Fig. 2C, D). Therefore, monoacylglycerols might inhibit biofilm formation in both *Y*. *enterocolitica* and *X*. *oryzae* by different biological mechanisms than it does in *S*. *mutans*.

Based on the results in Fig. 1, not all monoacylglycerols had inhibitory activity on biofilm formation. There were two types of inhibitory monoacylglycerols in the carbon skeleton length of the fatty acid moiety. One type of fatty acid moiety in monoacylglycerols was similar in carbon skeleton length to that of monolaurin, which consists of glycerol and lauric acid (C_12_), and the other was similar in carbon skeleton length to that of monobehenin, which consists of glycerol and behenic acid (C_22_). Such a specific and strong response of the carbon skeleton length suggests that the inhibition of biofilm formation was not simply due to the surfactant effect of monoacylglycerols and this suggestion was supported by no significant difference on the cell surface hydrophobicity change of *S*. *mutans* by monocaprin, monolaurin, and monocaprin (Additional file 1: Fig. 3).

Two specific carbon skeleton length regions for the inhibition of biofilm formation suggested two distinct response mechanisms to monoacylglycerols in bacteria. However, the existence of both response mechanisms in bacteria is strain specific. *A*. *hydrophila*, whose biofilm formation was not changed by monoacylglycerols, might not have any response mechanism. *S*. *mutans* and *X*. *oryzae*, whose biofilm formation was inhibited by molecules around both monolaurin and monobehenin, might possess both response mechanisms. *Y*. *enterocolitica*, whose biofilm formation was inhibited by molecules around only monobehenin, might have one corresponding response mechanism. The biological mechanism of each response mechanism for the inhibition of biofilm formation by monoacylglycerols is unknown. However, the preventive effect of monolaurin on caries in rats by reducing the CFU number of *S*. *mutans* in a previous study (Lynch et al. 1983) supports our observation.

**Fig. 3:**
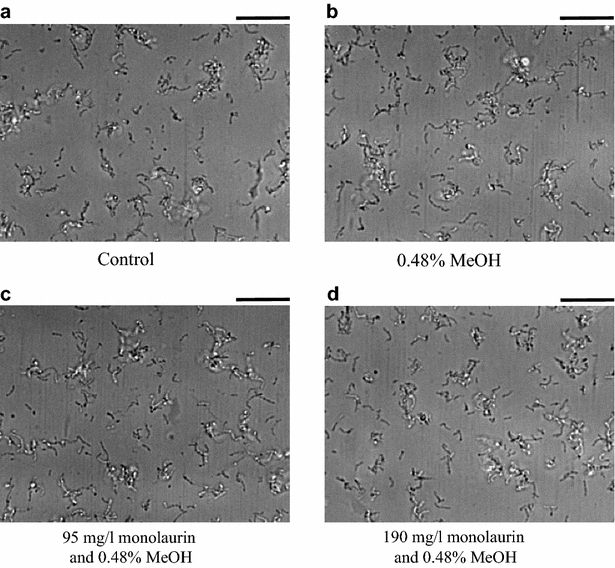
Biofilm formation of *S*. *mutans* in a continuous flow cell culture after a 1-h incubation in order to observe the initial attachment of cells. Cells were incubated in **a** BHI-S media, **b** BHI-S media with 0.48 % methanol, **c** BHI-S media with 0.48 % methanol and 95 mg/l monolaurin, and **d** BHI-S media with 0.48 % methanol and 190 mg/l monolaurin. The *scale bar* on the *top right* of pictures represents 20 μm

**Fig. 4:**
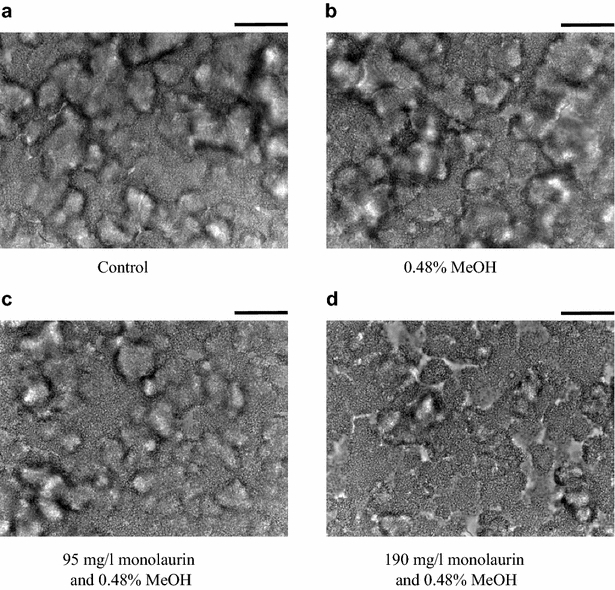
Biofilm formation of *S*. *mutans* in a continuous flow cell culture after a 24-h incubation in order to observe the biofilm development. Cells were incubated in **a** BHI-S media, **b** BHI-S media with 0.48 % methanol, **c** BHI-S media with 0.48 % methanol and 95 mg/l monolaurin, and **d** BHI-S media with 0.48 % methanol and 190 mg/l monolaurin. The *scale bar* on the *top right* of the images represents 40 μm

To test the inhibitory activity of monolaurin on biofilm formation in *S*. *mutans*, one of the continuous culture systems had been adapted and the biofilm structures were observed under a microscope (Figs. 3, 4). Because monolaurin was dissolved in methanol, the methanol effect was first observed as a control. There was no significant change in biofilm formation in 0.48 % methanol after either 1- or 24-h incubations (Figs. 3b, 4b, respectively). After a 1-h incubation, the initial attachment of *S*. *mutans* was indistinguishable from those of all four tested cultures (Fig. 3). However, biofilm formation after the 24-h incubation was inhibited significantly by 95 and 190 mg/l monolaurin (Fig. 4c, d). Increasing the monolaurin concentration inhibited biofilm formation in *S*. *mutans* more. Monolaurin is a surfactant and can be easily expected to inhibit biofilm formation in bacteria by changing cell’s hydrophobicity; however, monolaurin unexpectedly inhibited the development of biofilm instead of initial cell attachment. This observation solidified the suggestion that the inhibitory activity of monoacylglycerols was not due to their detergent characteristics but to changing cellular physiology by unknown response mechanisms for monoacylglycerols and therefore, biofilm formation as well. Additionally, the strong inhibition of monolaurin on biofilm formation in *S*. *mutans* suggests that monolaurin is the best oral hygiene compound among all tested monoacylglycerols.

## Conclusions

Monoacylglycerols inhibited the biofilm formation of three bacterial strains, *S*. *mutans*, *X*. *oryzae*, and *Y*. *enterocolitica*, in a strain specific manner with two specific lengths of fatty acid moiety, monolaurin and monobehenin. This result suggested that biofilm formation was not inhibited by the detergent characteristics of monoacylglycerols. This suggestion was supported by the inhibitory action of monolaurin on biofilm development but not on the initial cell attachment of *Y*. *enterocolitica* in flow cytometric observation.

## Additional file

10.1186/s40064-016-3182-5 **Figure S1**. Effect of monoacylglycerols on the biofilm formation of four bacterial strains, (**A**) *A. hydrophila*, (**B**) *S. mutans*, (**C**) *Y. enterocolitica*, and (**D**) *X. oryzae*. The biofilm amount was evaluated with 1 % crystal violet. Data points are the average of eight experiments and are presented as mean ± SD. The tested monoacylglycerols were monocaprylin (open square), monocaprin (closed square), monolaurin (open diamond), monomyristin (closed diamond), monopalmitin (open triangle), monostearin (closed triangle), monoarachidin (open circle ), and monobehenin (closed circle). **Figure S2**. Effect of monoacylglycerols on the cell growth of *S. mutans*. The relative cell density was evaluated by spectroscopic absorbance change at 595 nm. Data points are the average of eight experiments and are presented as mean ± SD. The tested monoacylglycerols were monocaprylin (open square), monocaprin (closed square), monolaurin (open diamond), monomyristin (closed diamond), monopalmitin (open triangle), monostearin (closed triangle), monoarachidin (open circle), and monobehenin (closed circle). Please note that the missing data when compared with Fig. S1 was due to the turbidity cause by insolubility of monoacylglycerols. **Figure S3**. Effect of monocaprin, monolaurin, and monomyristin on the hydrophobicity of cell surface of S. mutans. The experiment was performed according to a previous study (Infect Immun, 2004, 72(10): 6032–6039).
