# Supplementary material for: Inhibitory activity of monoacylglycerols on biofilm formation in Aeromonas hydrophila, Streptococcus mutans, Xanthomonas oryzae, and Yersinia enterocolitica
Source: Springerplus. 2016 Sep 9;5(1):1526. doi: 10.1186/s40064-016-3182-5 (PMC5017964; doi:10.1186/s40064-016-3182-5)
Supplement: Supplementary file 1 — 10.1186/s40064-016-3182-5 Figure S1. Effect of monoacylglycerols on the biofilm formation of four bacterial strains, (A) A. hydrophila, (B) S. mutans, (C) Y. enterocolitica, and (D) X. oryzae. The biofilm amount was evaluated with 1 % crystal violet. Data points are the average of eight experiments and are presented as mean ± SD. The tested monoacylglycerols were monocaprylin (open square), monocaprin (closed square), monolaurin (open diamond), monomyristin (closed diamond), monopalmitin (open triangle), monostearin (closed triangle), monoarachidin (open circle ), and monobehenin (closed circle). Figure S2. Effect of monoacylglycerols on the cell growth of S. mutans. The relative cell density was evaluated by spectroscopic absorbance change at 595 nm. Data points are the average of eight experiments and are presented as mean ± SD. The tested monoacylglycerols were monocaprylin (open square), monocaprin (closed square), monolaurin (open diamond), monomyristin (closed diamond), monopalmitin (open triangle), monostearin (closed triangle), monoarachidin (open circle), and monobehenin (closed circle). Please note that the missing data when compared with Fig. S1 was due to the turbidity cause by insolubility of monoacylglycerols. Figure S3. Effect of monocaprin, monolaurin, and monomyristin on the hydrophobicity of cell surface of S. mutans. The experiment was performed according to a previous study (Infect Immun, 2004, 72(10): 6032–6039). [file 40064_2016_3182_MOESM1_ESM.docx]

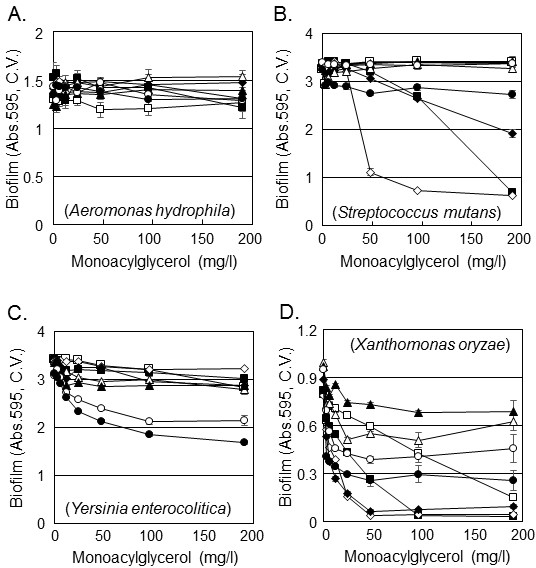


**Figure S1.** Effect of monoacylglycerols on the biofilm formation of four bacterial strains, (A) *A*. *hydrophila*, (B) *S*. *mutans*, (C) *Y*. *enterocolitica*, and (D) *X*. *oryzae*. The biofilm amount was evaluated with 1% crystal violet. Data points are the average of eight experiments and are presented as mean ± SD. The tested monoacylglycerols were monocaprylin (□), monocaprin (■), monolaurin (◇), monomyristin (◆), monopalmitin (△), monostearin (▲), monoarachidin (○), and monobehenin (●).


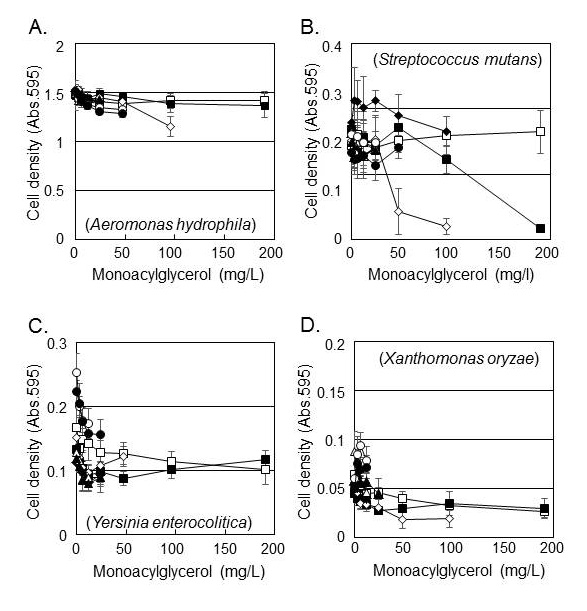


**Figure S2.** Effect of monoacylglycerols on the cell growth of *S*. *mutans*. The relative cell density was evaluated by spectroscopic absorbance change at 595 nm. Data points are the average of eight experiments and are presented as mean ± SD. The tested monoacylglycerols were monocaprylin (□), monocaprin (■), monolaurin (◇), monomyristin (◆), monopalmitin (△), monostearin (▲), monoarachidin (○), and monobehenin (●). Please note that the missing data when compared with Supplementary Fig. 1 was due to the turbidity cause by insolubility of monoacylglycerols.

**Figure S3.** Effect of monocaprin, monolaurin, and monomyristin on the hydrophobicity of cell surface of *S*. *mutans*. The experiment was performed according to a previous study (Infect Immun, 2004, **72**(10): 6032-6039).
